# Virtual Reality Exposure Therapy for Treating Social Anxiety: A Scoping Review of Treatment Designs and Adaptation to Stuttering

**DOI:** 10.3389/fdgth.2022.842460

**Published:** 2022-02-25

**Authors:** Ian Chard, Nejra van Zalk

**Affiliations:** Design Psychology Lab, Dyson School of Design Engineering, Imperial College London, London, United Kingdom

**Keywords:** social anxiety, social phobia, stuttering, stammering, virtual reality, VRET

## Abstract

Virtual Reality Exposure Therapy (VRET) has been shown to be an effective technique for reducing social anxiety. People who stutter are at greater risk of developing heightened social anxiety. Cognitive behavior therapy protocols have shown promise in reducing social anxiety in people who stutter, but no studies have investigated VRET targeting social anxiety associated with stuttering. The aim of the current review is to provide an overview of VRET techniques used to treat social anxiety and insights into how these techniques might be adopted in the case of comorbid stuttering and social anxiety. Twelve studies were reviewed to understand key distinctions in VRET protocols used to treat social anxiety. Distinctions include exercises targeting public speaking vs. general social anxiety, computer-generated virtual environments vs. 360° video, and therapist guided vs. automated VRET. Based on the review findings, we propose how certain features could be applied in the case of stuttering. Virtual therapists, inhibitory learning techniques and integration into speech therapy may be suitable ways to tailor VRET. Regardless of these different techniques, VRET should consider the situations and cognitive-behavioral processes that underlie the experience of social anxiety amongst people who stutter.

## Introduction

Stuttering is a developmental speech disorder characterized by the involuntary disruption to the fluent production of speech (1). Approximately 5% of people will experience stuttering in their lifetime, with roughly 1% prevalence within the overall population at any one time (2–4). Whilst many children who stutter recover naturally in early childhood, a significant proportion continue to stutter chronically (5).

A large body of evidence links stuttering to heightened levels of social anxiety (6, 7). Social anxiety is characterized as “*a marked, or intense, fear or anxiety of social situations in which the individual may be scrutinized by others*” [(1), p. 202]. For some people who stutter (PWS), this will manifest as subclinical social anxiety (or shyness) which is related but considered distinct from a clinical diagnosis of social anxiety disorder (SAD) (8). However, PWS are also at greater risk of developing SAD compared to fluent speakers. Approximately 46% of PWS are estimated to meet diagnostic criteria for SAD as opposed to 4% of fluent speakers (9). Core to the experience of social anxiety is the expectation of negative evaluation from others, and the overestimation of the consequences this will have (10). For PWS, social anxiety is embedded in speech and communication and revolves around the expectation that others will react negatively to one's stutter, and the overestimation of the consequences this will have (10, 11). This can result in greater speech dissatisfaction and avoidance of speaking situations (12, 13).

Social anxiety arises irrespective of speech fluency levels (12), therefore it is unsurprising that speech therapy techniques have limited effects on reducing social anxiety (14). It is therefore imperative that effective treatments be made available to PWS targeting social anxiety. PWS are likely to benefit from existing treatment protocols given their experience is rooted in the same principles of social anxiety such as fear of negative evaluation. However, tailoring treatment to consider the stuttering-specific nature of social anxiety will ensure treatment is as relevant as possible. As such, treatments should consider the cognitive processes, thoughts, and behaviors associated with social anxiety in stuttering (11), as well as situational factors and practical considerations. Cognitive Behavioral Therapy (CBT) is considered the gold-standard treatment for anxiety disorders (15) and several studies have investigated its use for PWS (16–18), with some adopting protocols which consider stuttering-specific processes (14, 19–24). This includes targeting speech-related fears, and the safety behaviors and expectations that are common amongst PWS. To the best of our knowledge, only three randomized trials have been conducted to date. In one study, CBT eliminated all SAD diagnoses in the sample and was associated with a decrease in social anxiety (14). Another study adopted a fully automated, online version of CBT, finding it was equally as effective as *in vivo* CBT at reducing social anxiety levels (23). The third trial also used automated CBT, demonstrating that when integrated into speech restructuring, it can aid the long-term maintenance of speech outcomes (22). Tailored CBT is therefore one appropriate method for targeting social anxiety associated with stuttering and may support retention of outcomes from speech therapy.

A central component of CBT is exposure therapy, a behavioral technique that involves immersing the patient in a scenario they fear, with the aim of adapting memories associated with feared stimuli using corrective information. Experiencing feared situations is considered key to the activation of mental representations in order to challenge unhelpful thoughts and promote new learning (25). This is usually done by confronting real feared stimuli (*in vivo*) or by visualizing them (imaginal). As the feared situation offers a strong forum for performing the cognitive elements of treatment, exposure is often considered as a standalone treatment (25), and some evidence suggests it is no less inferior than CBT (26).

Two separate theoretical models lay out alternative arguments regarding how exposure reduces anxiety and informs different treatment techniques. According to the *Emotional Processing Theory* (27), when corrective information is presented alongside the feared stimulus, it can overwrite the existing fear structure within memory. Within- and between-session habituation are the primary sources of this information. Because the reduction of anxiety runs counter to the expectations an individual has about their feared stimulus, they begin to associate the stimulus with a lower response. Thus, habituation is considered the cornerstone to successful anxiety reduction, determining progress through exercises. The *Inhibitory Learning Model* (28) suggests that a broader learning approach might explain how exposure operates. It purports that when patients are exposed to their feared stimulus along with corrective information, they learn how to inhibit the existing fear structure through learning new associations, rather than overwriting it. The primary technique associated with this theory is expectancy violation, in which exposure exercises are designed to violate the beliefs that patients may have about expected outcomes and promote the learning of new expectations. Both theoretical approaches have been widely used in exposure studies.

Exposure therapy may also be particularly relevant for stuttering. For example, the inhibitory learning model outlines strategies to deal with continued negative reactions that can occur post-treatment, and the often resulting reacquisition of anxiety (29). Exposure also provides the ideal medium to phase out stuttering-specific safety behaviors such as decreased verbal participation, avoidance of troublesome words and rehearsal of utterances before speaking (13). Exposure scenarios can be designed around these behaviors, so that PWS are forced to confront their feared situations which is necessary to learn new associations (29). Exposure is commonly used already in many speech and language programs. However, the general aim is different as it is used to practice new speech techniques in increasingly difficult situations.

In recent years, virtual reality (VR) has emerged as a promising tool to conduct exposure therapy. Virtual Reality Exposure Therapy (VRET) uses virtual environments to expose patients to anxiety-inducing stimuli. Typically, these stimuli are presented using a head-mounted display (HMD), which uses motion tracking and binocular graphics with a wide field of view to provide an immersive experience.

The ability of VR to create on-demand experiences has made it an appealing medium for exposure therapy, which requires repeated experience of fear-inducing situations. In the case of social anxiety, VRET involves the confrontation of social stimuli that provoke fear of public scrutiny and negative evaluation from others. Scenarios might include performative elements such as public speaking, or interactive elements such as speaking to those with authority and ordering food or drink. Recreating believable social interactions in a virtual environment is one of the greatest challenges for VRET, yet virtual social environments have proved to be effective in replicating human reactions to real social environments. Numerous studies have demonstrated heightened self-reported social anxiety and physiological responses when exposed to social environments in VR (30–34). Research has also showed that typical safety behaviors are observable in virtual environments (35, 36).

To date, there have been no robust assessments of exposure therapy for reducing social anxiety amongst PWS. Two studies begin to elucidate exposure's efficacy amongst PWS, one using an *in vivo* protocol (37) and the other using VRET (38). Participants in the *in vivo* study received 10 exposure sessions targeting public speaking fears, following the emotional processing approach. Exercises were also adapted to stuttering by including words designed to induce anxiety into the speeches. In the VRET study, participants received two exposure sessions targeting public speaking fears, but it is unclear whether this followed either the emotional processing or inhibitory learning approach to exposure. Additionally, participants were able to retreat to a “chill session” if they became too anxious which may have the inadvertent effect of teaching avoidant behaviors. Both studies claimed successful outcomes from exposure. Whilst the *in vivo* study did observe a reduction in social anxiety symptoms from pre- to post-treatment, this was not statistically analyzed. In addition, the VRET study lacked validated measures for social anxiety symptoms and did not provide outcome data. Despite the promising findings, methodological issues preclude conclusions regarding the effectiveness of stuttering-specific exposure therapy.

However, there is more robust evidence backing the use of VRET for social anxiety in the wider population. Three meta-analyses have examined the effects of VRET on social anxiety (39–41). Several others have examined the use of VRET more generally in treating various anxiety disorders, including SAD (42–45). Whilst these meta-analyses provide a helpful indicator of VRET's efficacy, they do not expand on the different approaches adopted in treatment protocols, and the key distinctions of treatment and study designs. One of the above meta-analyses covers some of these distinctions in their analyses (41). Findings showed that both standalone VRET and VRET integrated into psychotherapy were associated with improved treatment outcome. Similarly, VRET was effective for both participants with a SAD diagnosis, or a diagnosis of public speaking anxiety. However, many questions remain regarding the implementation of VRET and the processes that potentially make it effective. Analyses also covered the influence of several factors including number of VR sessions, total number of sessions, and ratio of VR sessions to total sessions, demonstrating no influence of these factors. Due to increasingly sophisticated technology, VRET can take many shapes and forms. This includes using smartphone-based VR (46, 47), 360° video for virtual environments (46–48), and virtual therapists (48). Yet no review paper comprehensively covers the key differences in treatment design. Understanding the comparative value of different techniques adopted in VRET is thus necessary to keep improving treatment design.

Given the suitability of exposure for PWS, the growth of VRET research, and the research gap concerning stuttering, it is important to consider how best to adapt treatment techniques for this population. The aim of the current study is therefore to provide further insights into how VRET might be adopted in the case of comorbid stuttering and social anxiety. Nevertheless, there is limited systematic research on the various techniques and approaches adopted in VRET protocols targeting social anxiety. One objective is therefore to provide a comprehensive review of the different approaches and techniques used in VRET design for adults. Although it would be useful to investigate VRET for non-adult populations, the child and adolescent literature is relatively limited, and developmental trajectories of social anxiety throughout childhood and adolescence preclude using the same CBT treatment protocols for adults and children who stutter [see e.g., (19) and (24) for adolescent and adult protocols, respectively], making comparisons difficult. Findings from this scoping review will outline distinctions in treatment designs adopted in adult VRET trials, and key findings from these studies. These approaches will be discussed in the context of stuttering. Whilst no rigorous trials have explored VRET for reducing social anxiety associated with stuttering, this paper will build on the knowledge gained from previous work and discuss what techniques are likely to be particularly suited to PWS.

## Methods

### Scoping Review

Given the acceleration of VRET research targeting social anxiety, a scoping review was chosen for the current paper to provide the information needed to conduct a qualitative synthesis of relevant literature, and to discuss the comparative value of different VRET techniques including considerations for adopting these for stuttering. The Preferred Reporting Items for Systematic reviews and Meta-Analyses extension for Scoping Review (PRISMA-ScR) guidelines were followed (49). No review protocol was created for this review.

### Search Strategy

Systematic literature searches were performed on September 22, 2021, using three databases: Web of Science, Scopus, and PsycINFO/PsycARTICLES. Search terms were primarily aimed at finding VRET trials targeting social anxiety, but also any possible developments in exposure related to stuttering. For each database, three searches were conducted. First, [(Virtual Reality OR VR) AND (Social anxiety OR social phobia OR social anxiety disorder OR SAD) AND (Therapy OR Treatment)]. Second, [(Virtual Reality OR VR) AND (Stutter^*^ OR Stammer^*^) AND (Therapy OR Treatment)]. Third, [Exposure AND (Therapy OR Treatment) AND (Stutter^*^ OR Stammer^*^)]. Supplementary material for complete search strategy using PsycINFO/PsycARTICLES is provided in Appendix A and was adapted for all database searches. Relevant studies identified in previous literature searches unrelated to the current scoping review were also included.

### Eligibility

Studies were included if they met the following eligibility criteria: (1) study used VRET aimed at reducing social anxiety symptoms, (2) study design included at least one comparison condition such as *in vivo* exposure/waitlist control, in addition to the experimental VRET condition, (3) comparison condition did not include VRET, (4) VRET was not combined with any medication, (5) study participants were adults, (6) study used random/quasi-random assignment/participant matching, (7) study used at least one validated and reliable measure of social anxiety symptoms, (8) report included sufficient statistical analysis—means/SD for each group, (9) study was published in a peer-reviewed journal article (no book chapters or dissertations), (10) study was published in English, and (11) full-text was available. Date of publication was not considered in the eligibility criteria so that all VRET protocols in the context of social anxiety were considered.

### Study Selection

**Figure 1:**
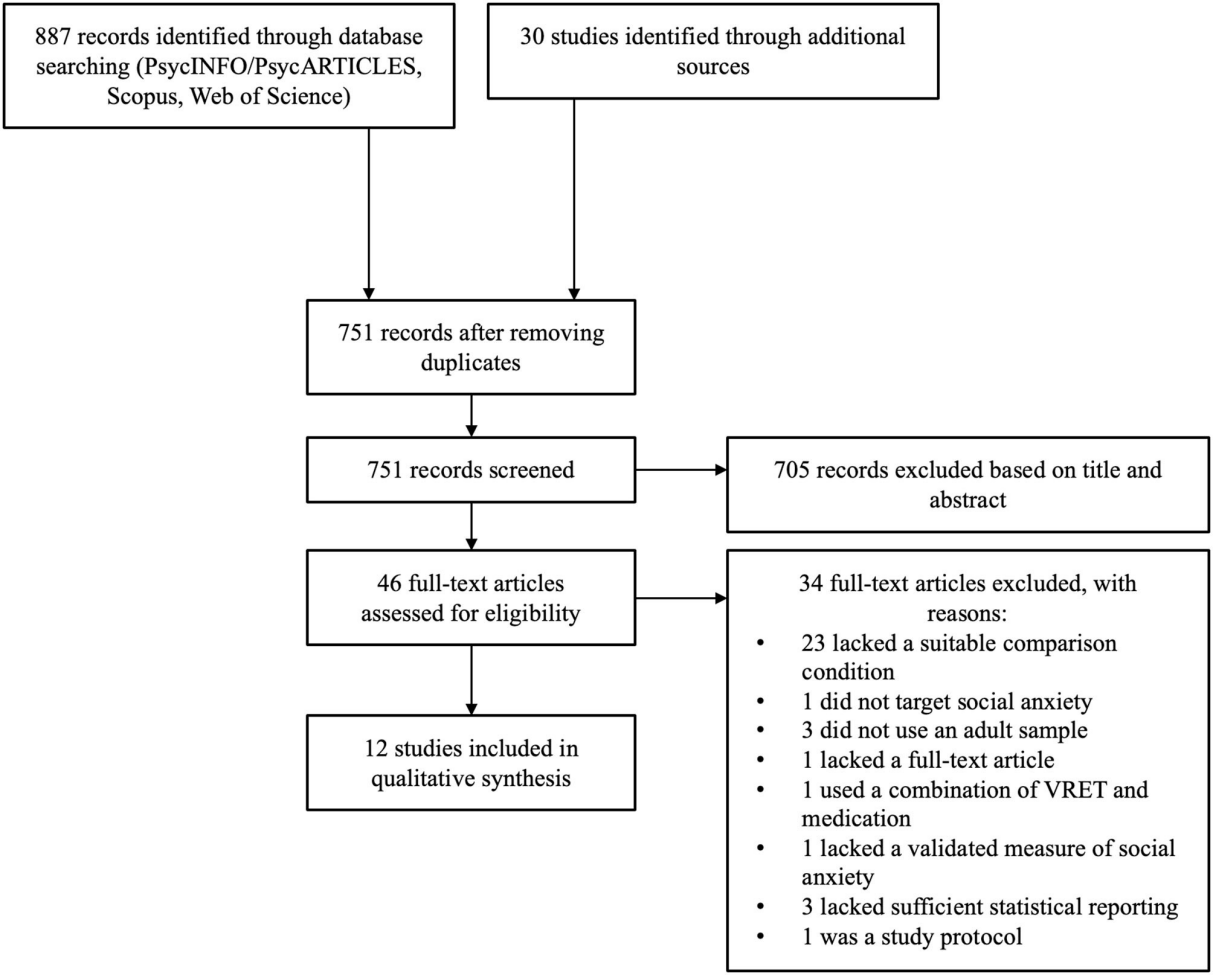
PRISMA-ScR diagram.

The first author was responsible for screening each record retrieved. Literature search results were initially exported into EndNote, and duplicates were deleted. Primary screening included reviewing the titles and abstracts of all results. Those which were irrelevant or did not fit eligibility criteria were excluded at this stage. Studies were then reviewed as part of secondary screening for thorough assessment against eligibility criteria. The reason for excluding each study not meeting criteria was noted. A full breakdown of this process is outlined in the PRISMA flowchart (Figure 1).

### Data Extraction

The data were obtained and summarized by the first author. A data extraction table was generated to note key features of VRET protocols for each eligible study. The following data were extracted: (1) authors and publication date, (2) sample size and no. of participants in each condition, (3) type of participant assignment, (4) mean age, (5) whether participants reported clinical/subclinical level of social anxiety, (6) social anxiety measures, (7) type of treatment/control used in comparison condition(s), (8) target of VRET (social anxiety or public speaking anxiety), (9) whether VRET was standalone integrated into CBT, (10) whether VRET followed emotional processing or inhibitory learning approach to exposure, (11) number of treatment sessions, (12) time from pre- to post-treatment (and follow-up if included), (13) whether VRET was delivered remotely or in-person, (14) whether there was a real or virtual therapist, (15) whether virtual environments were computer-generated or 360° video, (16) whether VRET was delivered using a HMD or smartphone HMD, and (17) whether analyses were based on completers or intention to treat. For each eligible paper, details of the treatment protocol, and key findings were also noted.

## Results: Key Distinctions in Vret Studies Targeting Social Anxiety

A total of 12 studies were eligible for this scoping review following the screening process. Table 1 provides a summary of the findings from this review. Studies were conducted in the USA (4), Canada (2), Israel (2), The Netherlands (1), France (1), Sweden (1) and the UK (1). Studies were published over a 19-year period between 2002 and 2021, with the quality of VR equipment varying across the papers. The mean age of participants ranged between 23 and 42 years but was skewed toward younger adults. Key distinctions in treatment protocols that have been adopted in these studies are outlined in more detail below and in Table 2.

**Table 1:** Studies included in scoping review, methodological details, and key features of VRET protocols.

**References**	**Participant age (*M*)**	**VRET (N)**	**Active control**	**Waitlist control**	**Comparison condition**	**Main variables of interest**	**Clinical diagnosis required**	**Target of VRET**	**Exposure technique**	**Type of virtual environment**	**Facial expressions in audience**	**HMD type**	**Therapist type**	**Integration into CBT**
Anderson et al. (50)	39	30	39	28	IVET, WL	FNE-B, PRCS, peak anxiety during speech, speech length	Yes	PSA	Unknown	Computer-generated	Yes	Unknown	Real	Yes
Anderson et al. (51)	42	13	15		IVET	FNE-B, PRCS, peak anxiety during speech, speech length	Yes	PSA	Unknown	Computer-generated	Yes	Unknown	Real	Yes
Bouchard et al. (52)	34.5	17	22	20	CBT-IV, WL	LSAS-SR, SPS, SIAS, FNE, BAT (SPRS)	Yes	General SA	Inhibitory learning	Computer-generated	Yes	Research-grade HMD	Real	Yes
Harris et al. (53)	Unknown	8		6	WL	LSAS, PRCS, STAI, ATPS, HR during voice sample	No	PSA	Unknown	Unknown	Unknown	Research-grade HMD	Real	No
Kampmann et al. (39, 54)	36.9	20	20	20	IVET, WL	LSAS-SR, FNE-B, speech duration, speech performance	Yes	General SA	Emotional processing	Computer-generated	Unknown	Research-grade HMD	Real	No
Klinger et al. (55)	32	18	18		CBT-IV	LSAS, RAS	Yes	General SA	Unknown	Computer-generated	Yes	Monitor	Real	Unknown
Lindner et al. (47)	31.4	25		25	WL	PSAS, LSAS-SR, FNE-B	Yes	PSA	Inhibitory learning	360° video	Unknown	Smartphone HMD	Real	No
Reeves et al. (46)	26.1	17 (Audience), 16 (Empty room)		18	WL	PSAS, LSAS-SR, FNE-B, speech duration	No	PSA	Unknown	360° video	Yes	Smartphone HMD	None	No
Robillard et al. (56)	34.9	14	16	15	CBT-IV, WL	LSAS, SPS, ASC-P, ASC-C, FNE	Yes	General SA	Unknown	Computer-generated	Yes	Research-grade HMD	Real	Yes
Safir et al. (57)	27	25	24		CBT-IV	LSAS, SSPS, FNE, self- and observer-rated anxiety during speech	No	PSA	Unknown	Computer-generated	Unknown	Research-grade HMD	Real	Yes
Wallach et al. (58)	27	28	30	30	CBT-IV, WL	LSAS, SSPS, FNE, self- and observer-rated anxiety during speech	No	PSA	Unknown	Computer-generated	Unknown	Research-grade HMD	Real	Yes
Zainal et al. (48)	23.3	26		18	WL	SPDQ, SIAS, MASI	Yes	General SA	Emotional processing	360° video	Unknown	Consumer HMD	Virtual	No

*IVET, in vivo exposure therapy; WL, waitlist; CBT-IV, CBT with in vivo exposure therapy; FNE-B, fear of negative evaluation—brief form (59); PRCS, personal report of confidence as a speaker (60); LSAS-SR, Liebowitz social anxiety scale—self reported version (61); SPS, social phobia scale (62); SIAS, social interaction anxiety scale (62); FNE, fear of negative evaluation (63); BAT, behavioral assessment task; SPRS, social performance rating scale (64); LSAS, Liebowitz social anxiety scale (65); RAS, Rathus assertiveness schedule (66); PSAS, public speaking anxiety scale (67); ASC-P, appraisal of social concerns—probability subscale (68); ASC-C, appraisal of social concerns—consequences subscale (68); SSPS, self-statements during public speaking (69); SPDQ, social phobia diagnostic questionnaire (70); MASI, measure of anxiety in selection interviews (71); PSA, public speaking anxiety; SA, social anxiety*.

**Table 2:** Key features of studies included in the scoping review.

**Study**	**Key features**
Anderson et al. (50, 51)	• Participants had a clinical diagnosis of SAD • Exercises targeted public speaking anxiety using a virtual conference room (~5 audience members), a virtual classroom (~35 audience members), and a virtual auditorium (100+ audience members). Scenario, audience reactions, and task details were adapted in accordance with participant's fear hierarchy • Exposure techniques not specified • Computer-generated virtual environments • Virtual avatars displayed facial expressions • HMD type not specified • Assisted by therapist–2 clinical psychologists, 3 doctoral students • VRET integrated into CBT
Bouchard et al. (52)	• Participants had a clinical diagnosis of SAD • Exercises targeted general social anxiety using a public speaking scenario, dinner party, busy café and shoe store targeting performance, intimacy, scrutiny, and assertiveness anxiety respectively • Inhibitory learning approach • Computer-generated virtual environments—low-fidelity • Virtual avatars displayed facial expressions • Research-grade HMD—eMagin z800 • Assisted by therapist—doctoral students with 1-year CBT experience • VRET integrated into CBT
Harris et al. (53)	• Participants did not have a clinical diagnosis of SAD, but had elevated levels of social anxiety • Exercises targeted public speaking anxiety using an auditorium. Exercises were made increasingly difficult through larger audiences and audience members talking and asking the participant to speak louder • Exposure techniques not specified • Type of virtual environment not specified • Use of facial expressions not specified • Research-grade HMD—Virtual–I/0 • Assisted by therapist—clinical psychologist • VRET delivered as a standalone treatment
Kampmann et al. (54)	• Participants had a clinical diagnosis of SAD • Exercises targeted general social anxiety and placed an emphasis on verbal interaction. Exercises were based on either one-to-one or group scenarios and included giving a talk in front of an audience followed by questions from the audience, talking to a stranger, buying and returning clothes, attending a job interview, being interviewed by journalists, dining in a restaurant with a friend, and having a blind date • Emotional processing approach—participants rated their anxiety level before, during and after each exercise, and repeated it another time if anxiety did not decrease • Computer-generated virtual environments—high-fidelity • Use of facial expressions not specified • Research-grade HMD—nVisor SX • Assisted by therapist—clinical psychologists, clinical psychology master's degree students • VRET delivered as a standalone treatment
Klinger et al. (55)	• Participants had a clinical diagnosis of SAD • Exercises targeted general social anxiety using a public speaking scenario, dinner party, busy café and shoe store targeting performance, intimacy, scrutiny, and assertiveness anxiety respectively • Exposure techniques not specified • Computer-generated virtual environments—low-fidelity • Virtual avatars displayed facial expressions • Exposure conducted using a computer monitor • Assisted by therapist—clinical psychologist • Unknown whether VRET was integrated into CBT
Lindner et al. (47)	• Participants had clinical levels of public speaking anxiety • Exercises targeted public speaking anxiety using an auditorium, wedding reception and meeting room. Audience size and task difficulty was modified throughout exercises. • Inhibitory learning approach • 360° video virtual environments • Use of facial expressions not specified • Smartphone-based HMD—Samsung Gear VR • Assisted by therapist-−1 clinical psychologist, 3 clinical psychology master's degree students • VRET delivered as a standalone treatment
Reeves et al. (46)	• Participants had subclinical levels of social anxiety • Exercises targeted public speaking anxiety, but participants spoke either in front of an audience or an empty room. In both conditions, the room size increased throughout exercises. In the audience condition, audience size also increased. • Exposure techniques not specified
	• 360° video virtual environments • Virtual avatars displayed facial expressions • Smartphone-based HMD—Samsung Gear VR • No therapist • VRET delivered as a standalone treatment
Robillard et al. (56)	• Participants had subclinical levels of public speaking anxiety • Exercises targeted general social anxiety using a public speaking scenario, dinner party, busy café and shoe store targeting performance, intimacy, scrutiny, and assertiveness anxiety respectively • Exposure techniques not specified • Computer-generated virtual environments—low-fidelity • Virtual avatars displayed facial expressions • Research-grade HMD—eMagin z800 • Assisted by therapist—training not specified • VRET integrated into CBT
Safir et al. (57) and Wallach et al. (58)	• Participants had subclinical levels of public speaking anxiety • Exercises targeted public speaking anxiety using a large audience. Audience reactions and task details were adapted in accordance with participant's fear hierarchy • Exposure techniques not specified • Computer-generated virtual environments • Use of facial expressions not specified • Research-grade HMD—VFX3D • Assisted by therapist—clinical psychology students • VRET integrated into CBT
Zainal et al. (48)	• Participants had a clinical diagnosis of SAD • Exercises targeted general social anxiety using a formal job interview and informal dinner party. Performance, intimacy, assertiveness, and observation anxiety were targeted. • Emotional processing approach—participants moved onto the next exposure exercise if anxiety had decreased by 50% across three consecutive attempts or whether ratings before an attempt were sufficiently low • 360° video virtual environments • Use of facial expressions not specified • Consumer HMD—Pico Goblin • Assisted by virtual therapist—used voiceover to outline principles of exposure, provide instructions and to coach participants through exercises • VRET delivered as a standalone treatment

### Clinical vs. Subclinical Social Anxiety

All the studies in this analysis target adults with elevated social anxiety levels and discuss the relevance of their findings within a clinical context. However, whilst some include a clinical diagnosis of SAD or public speaking anxiety in their eligibility criteria (47, 48, 50–52, 54–56), some only use elevated social anxiety (46, 53, 57, 58). The investigation of non-clinical samples is likely to become more important with the rise of self-guided, remote treatments that are not designed as clinical interventions, but as accessible treatments for subclinical use.

Both VRET protocols targeting clinical and subclinical levels of social anxiety are likely to have suitable applications for stuttering. Clinical VRET could be useful for the significant subset of PWS who have a diagnosis of SAD (9), whilst generally elevated levels of social anxiety (6, 7) may make subclinical VRET an appealing option for some. However, subclinical VRET may be particularly suited for delivery alongside speech therapy. Some authors have suggested that all PWS receiving speech therapy should also receive psychological treatment (23), given findings that mental health conditions can disrupt the progress made from speech therapy (72). Such is the highly entwined nature of comorbid stuttering and social anxiety, that cognitive and behavioral change are considered necessary for managing stuttering (73). Clinical VRET is unlikely to be appropriate in this context but a less substantial form of VRET targeting subclinical social anxiety may be more suited. Integrating subclinical VRET into speech therapy may also be one strategy to increase the uptake of social anxiety treatment amongst PWS, given this is often the first port of call. VRET is also far simpler and cheaper to administer than *in vivo* exposure, which could reduce the burden of delivering additional treatment.

### Public Speaking vs. General Social Anxiety

Public speaking fears were targeted in 7 of the 12 studies (46, 47, 50, 51, 53, 57, 58). Given that a significant proportion of people only experience performance-only social anxiety (74), it is unsurprising that most studies target these scenarios only. Performance scenarios are easier to develop and conduct as they do not include interactions and therefore remove the need for turn-taking conversation that can be difficult to recreate realistically in VRET.

The earliest of these studies (53) used an auditorium scenario and made tasks increasingly difficult as participants progressed. This involved increasingly larger audiences and audience members talking and asking the participant to speak louder. VRET was associated with a decrease in social anxiety scores, but this was not true for all measures. At post-treatment, there was little difference between the VRET and waitlist conditions. A more recent study also manipulated the setting for the public speaking task (47). Using an auditorium, wedding reception and meeting room, they changed the size of the audience and the difficulty of the task. Findings were more encouraging, showing a significantly greater decrease on all social anxiety measures compared to the waitlist condition. Two studies adapted public speaking tasks to the individual, changing the scenario, audience reactions, and task details in accordance with each participant's fear hierarchy (50, 58). Both studies showed comparable reductions on most social anxiety measures compared to *in vivo* exposure therapy. However, they also showed that VRET did not reduce fear of negative evaluation to the same extent as *in vivo* exposure therapy. Follow-up assessments of participants in these studies showed treatment gains were maintained long-term (51, 57). Another recent study further explored how the nature of the public speaking task might influence treatment efficacy by comparing VRET protocols using audiences or empty rooms (46). In both conditions, the room size increased throughout sessions, and the audience size also grew in the audience condition. Both treatments were effective across all measures; however, the empty room version failed to show superiority over waitlist with regards to fear of negative evaluation. Together, these findings support the efficacy of VRET targeting public speaking fears, but some protocols are limited in reducing fear of negative evaluation, a core element of social anxiety. Inclusion of social cues is likely necessary for this (46), however cannot explain the null findings from other studies (50, 58).

Whilst individuals experiencing social anxiety more generally are likely to experience public speaking anxiety and may benefit somewhat from the above protocols, a more varied treatment protocol targeting a variety of scenarios is more appropriate. According to the *Diagnostic and Statistical Manual of Mental Disorders* (DSM-5), the “Performance only” subtype is considered distinct from general SAD (1). Targeting feared stimuli in multiple contexts is also considered key for maximizing exposure according to the inhibitory learning theory (29). The other five studies that were reviewed targeted a variety of social evaluative scenarios (48, 52, 54–56).

All studies used environments targeting different facets of social anxiety such as performance, intimacy, assertiveness, scrutiny, and observation anxiety. Three of these studies adopted the same set of stimuli (52, 55, 56). Exposure exercises replicated a public speaking scenario, dinner party, busy café and shoe store targeting performance, intimacy, scrutiny, and assertiveness anxiety respectively. Treatment gains from these protocols were shown to be superior to waitlist condition (52, 56) and comparable to *in vivo* exposure therapy (52, 55). One of the studies also found superiority of VRET over *in vivo* exposure at both post-treatment and 6-month follow-up on Liebowitz Social Anxiety Scale and Social Phobia Scale scores (52). Another study only used two scenarios (formal job interview and informal dinner party) but the content changed between exercises to target different domains of social anxiety including performance, intimacy, assertiveness, and observation anxiety (48). Again, treatment gains from VRET were found to be superior to the waitlist condition on all measures. The last of these studies placed an emphasis on verbal interaction through their scenarios, noting previous protocols had been limited (54). This is important for VRET targeting social anxiety more generally, given that verbal interaction forms a central part of social fears. Participants were exposed to a variety of environments that were based on either one-to-one or group scenarios. Whilst VRET decreased social anxiety significantly compared to waitlist, and was comparable with *in vivo* exposure, VRET failed to reduce fear of negative evaluation. Given the mixed findings regarding fear of negative evaluation, it is unlikely that the domains of social anxiety that VRET targets influence the reduction of this construct.

VRET targeting general social fears will be more appropriate for the majority of PWS. Findings from a recent preprint suggest that, besides speaking on the phone, situational factors influencing social anxiety were largely similar in PWS and fluent speakers, including those with SAD (75). Findings from another preprint also showed that general speech-related fears are common across PWS (76). VRET targeting a variety of social anxiety domains is therefore likely to have a more efficacious outcome than performance only VRET. This is supported by findings suggesting ~22–46% of PWS meet diagnostic criteria for SAD (9, 77). However, there are some situational factors that should be considered when designing VRET protocols. Findings from both of the above preprints suggest that fear of speaking on the telephone is particularly elevated amongst PWS and may constitute a distinguishable sub-type of social anxiety amongst PWS (75, 76). A telephone-only treatment may therefore be appropriate for some PWS. However, as the nature of social anxiety has been shown to vary across PWS, one challenge will be to adapt VRET to individual needs.

Other than situational factors, stuttering-specific cognitive-behavioral processes should also be considered in VRET design. Thoughts and expectations are likely to revolve around speech and the perceived costs that stuttering will have (78). Some examples include “*People focus on every word I say*,” “*Everyone in the room will hear me stutter*,” and “*No one will like me if I stutter*” (79). Other cognitive and behavioral mechanisms which maintain social anxiety are also modified for the experience of stuttering (11). For example, PWS will often use stuttering-specific safety behaviors such as avoiding and substituting feared words, avoiding anxiety-inducing communicative situations, and rehearsing speech before speaking (13, 80). VRET protocols designed for PWS need to consider how social anxiety and the mechanisms that underpin it will differ to ensure maximum relevance of treatment.

### Emotional Processing vs. Inhibitory Learning

Not all studies specified the details of how they conducted exposure, and more specifically what the aim of exposure exercises were. However, there were studies adopting both emotional processing and inhibitory learning methodologies. Two of the studies adopted the emotional processing method (48, 54). This involves targeting anxiety levels throughout exercises with a focus on habituation and gradual exposure to increasingly anxiety-inducing situations in line with an individual's fear hierarchy. Participants in one of these studies rated their anxiety levels before, during and after each exercise, and repeated it another time if anxiety did not decrease (54). Participants in the other study moved onto the next exposure exercise if anxiety had decreased by 50% across three consecutive attempts or whether ratings before an attempt were sufficiently low (48). The benefit of this approach is that is simple to administer and therefore could have greater potential for delivering VRET at scale.

Two other studies used the inhibitory learning model to inform their treatment design (47, 52). Therefore, the aim of these studies was to target participant expectations to develop new non-threatening associations with feared social situations. These kinds of techniques may be well-suited to general social anxiety since it can arise in various situations across varying contexts. Inhibitory learning is proposed as a method to inform new learning of associations across contexts rather than the specific context in which exposure was completed. Thus, varying the context for exposure is also considered important to broaden that learning.

Inhibitory learning may be well-suited to dealing with the negative reactions that are likely to continue post-treatment. At the heart of social anxiety lies the fear of negative evaluation that involves the assumptions of being negatively evaluated, and the overestimation of the consequences this will have. Models of social anxiety suggest that limitations in social performance, which often causes negative evaluation, are a consequence of anxiety (10, 81). However, the speech of someone who stutters can be the source of continued negative evaluation throughout life, meaning reconciling these expectations is made particularly difficult. These events may confirm existing expectations, and clash with newly learnt associations. Whilst presenting corrective information is the fundamental method of exposure therapy, the inhibitory learning theory suggests occasionally displaying one's feared expectation can be used as a way of learning how to deal with negative outcomes, and to attenuate subsequent reacquisition of fear (29). The stuttering-specific nature of social anxiety also needs to be considered in this approach. For example, protocols should consider how expectations and safety behaviors will differ amongst PWS and adapt exercises accordingly. The therapist should also emphasize that exposure is targeting expectations and beliefs regarding others' reactions to speech/social performance, rather than expectations of stuttering itself.

### Computer-Generated vs. 360° Video

Virtual environments can be created in two ways: computer-generated or using 360° video. Most of the studies used computer-generated environments (50–52, 54–58). This involves environments replicated using software, and computer-generated avatars as people. Some of the older studies used extremely rudimentary environments (52, 55, 56) involving 2D images overlaid onto low-fidelity backgrounds and less realistic lighting compared to more recent studies (54). This progression in technology has meant greater realism within virtual environments, and scenes can now be manipulated and controlled easily. In the case of VRET for social anxiety, the primary benefit is that the reactions of others in the scene can be controlled. Users are also able to interact with objects within the scene using controllers. Despite this progression, older studies using environments with low levels of realism, have still shown successful reduction of social anxiety (52, 55, 56).

Recent advances in camera technology have also led to the emerging field of VRET research using 360° video. Three of the studies in this analysis used this method for creating their virtual environments (46–48). These environments use a spherical video recording of a real environment simulating an anxiety-inducing situation, which is then played back in VR for the purposes of exposure. One of the suggested benefits of this medium is that it is more realistic than computer-generated environments. It is also cheaper and does not require an experienced programmer to create environments.

The concept of presence is key for understanding the comparative value of each of these methods. Presence is defined as the “perceptual illusion” of being physically present and involved within a virtual environment (82). It is also conceptualized as a construct that allows fear to be experienced toward a virtual stimulus (83). Thus, according to the emotional processing theory, presence is important because activation of the fear structure is necessary for successful exposure. Presence is likely to be influential under the inhibitory learning model as well. Greater presence indicates greater involvement with the scene which is necessary to attend to anxiety-inducing stimuli for the purpose of extinction learning. Realism does influence presence, however not as much as other factors such as tracking level, stereoscopy, and field of view (84). Recent evidence suggests that computer-generated and 360° video environments are comparable in terms of presence and ability to induce anxiety (85), suggesting differences in realism did not have an effect. However, this study used passive environments including little social interaction. The main limitation of 360° video is that users are limited to passive navigation around the environment. This is fine for public-speaking scenarios but can be particularly challenging for developing social environments involving interaction and turn-taking. Controlling the environment based on the user's behavior is possible within computer-generated environments, generating greater agency and intentionality which are theorized to influence presence (86). Findings have also shown that whilst presence does not influence VRET outcome in the treatment of social anxiety, the involvement factor does (87). Both methods have shown sufficient presence levels and have been used to successfully reduce levels of social anxiety in VRET. However, computer-generated environments may be more suited to targeting social interaction as part of social anxiety treatment.

### Facial Expressions

Most studies manipulated audience and avatars' facial expressions to vary task difficulty (46, 50–52, 55, 56). This is a common approach to targeting fear of negative evaluation, particularly within public-speaking scenarios. However, it is unclear how effective these VRET protocols have been at reducing fear of negative evaluation. Some of these studies have shown superiority of VRET over waitlist control (46, 52, 56), but this is not the case for all studies (50). The absence of facial expressions in another study's VRET protocol were suggested to potentially contribute to a lack of fear of negative evaluation effect, whilst *in vivo* exposure did successfully reduce it (54). The findings suggest standalone exposure can target fear of negative evaluation, but factors within the virtual presentation disrupted this. The authors suggest that facial expressions may be more influential in one-to-one interactions than in audience settings, like those used in the above study reporting null findings (50), where they may be more difficult to register.

The use of 360° video may support the reduction of fear of negative evaluation through presenting clearer images of facial expressions. Findings from one study showed that exposure to a virtual audience led to significantly greater reductions in fear of negative evaluation than exposure to an empty room (46). This suggests that the inclusion of fear-relevant stimuli, including facial expressions, benefits fear of negative evaluation reduction, even in a public speaking context. The authors suggest that using 360° video meant audience faces were easier to register. Thus, the inclusion of facial expressions is likely to be an important factor for targeting fear of negative evaluation, so long as faces can be registered. In computer-generated environments, this may be more challenging for public-speaking contexts due to limitations in computer graphics.

Facial feedback is also likely to be an important factor for PWS. However, exposure to positive feedback may be particularly advantageous given PWS tend to avoid positive feedback from others to a greater extent than fluent speakers (88). Exposure to scenarios including positive feedback are commonplace, especially in protocols adopting the inhibitory learning method as this aims to teach new associations with such situations.

### HMD Type

The type of HMD used in VRET will be highly influential in determining how immersive exposure exercises are. HMDs have also substantially improved across the last few decades to include wider field of view, higher-definition images, and controllers to interact with virtual environments. These are factors known to influence presence (84), making them likely to improve VRET outcomes. One of the earlier studies in this review did not use a HMD at all, displaying the virtual environments to participants on a computer screen (55). Participants moved around the environments using a mouse. However, this is now uncommon given the availability of HMDs which provide a far more immersive experience.

Most of the studies used older research-grade HMDs, such as the eMagin z800 and nVisor SX, which are not available for consumer use (52–54, 56–58). However, this is no longer necessary given the quality and price of consumer HMDs. The Pico Goblin used by one of the more recent studies has a wider field of view and greater screen resolution than all of the above HMDs (48). Traditionally, HMDs have needed computers to provide the computational power required for VR. However, the Pico Goblin is one example of a standalone HMD which does not require this. The commercialization of VR technology has made this equipment more accessible both to researchers and users.

Further development in smartphone capabilities mean they are also powerful enough to run VR programs as a standalone HMD. When paired with a smartphone-based HMD, it displays an image to each eye through the phone's screen to create an immersive experience. The phone's gyroscope controls head movement around the scene. Two studies used smartphone HMDs (46, 47). Both used the Samsung Gear VR HMD with Samsung smartphones. One of these studies also used a public speaking app to conduct exposure exercises (47). Findings suggest smartphone VRET can be effective at reducing social anxiety, opening up the prospect of any individual owning a VR device to conduct VRET.

All HMDs have demonstrated suitability for delivering VRET targeting social anxiety. More modern HMDs have the added benefit of higher specs which may contribute to greater presence (e.g., interaction using controllers). Smartphone HMDs are not currently at the same standard but are becoming increasingly sophisticated. The choice of HMD will likely depend on factors such as budget and delivery of treatment (e.g., remote vs. in-person).

### Therapist vs. Virtual Therapist

Therapeutic alliance refers to the extent of collaboration and purposeful action between therapist and patient (89) and is considered key for treatment success. The role of the therapist is to provide encouragement, guidance through treatment exercises, and to develop the patient's trust and investment in the treatment process (89). However, inconsistent findings suggest the influence of therapeutic alliance on treatment outcome is more complicated in a social anxiety context (90). Some scholars suggest that as fear of negative evaluation is a core element of social anxiety, the interpersonal relationship a patient has with their therapist might itself induce anxiety which disrupts the link to symptom change (91).

A therapist was used in most studies (47, 50–58). The level of training varied between studies, with some using clinical psychology students (52, 57, 58), some using trained clinical psychologists (53, 55), and some using a combination (47, 50, 51, 54). Responsibilities included guiding the patient through treatment, manipulating tasks according to individual fears, helping to process emotions and thoughts during exposure exercises, and providing encouragement. One study found that therapeutic alliance was a significant predictor of later symptom change in participants receiving VRET (52). Another study also found no difference in therapeutic alliance levels between the VRET and *in vivo* exposure conditions (50). These findings therefore suggest therapeutic alliance may play a more influential role in the treatment of social anxiety when using VRET.

One study included no therapist (46). A researcher was present throughout exposure exercises, but they dealt with the practicalities of administering exposure exercises. It is unclear what guidance participants received and whether other traditional therapist responsibilities were covered in other ways. This approach is uncommon, and research suggests the lack of therapist contact can result in lower treatment efficacy and adherence to treatment (92, 93).

More recent research has investigated eliminating the therapist and replicating them virtually, aided by the development of standalone and smartphone HMDs. Embodied conversational agents can either appear visually or provide guidance through audio (94). The benefit of such self-guided treatments is that they are likely to be far more scalable than existing VRET protocols (95). This approach may also be particularly suitable for treating social anxiety as it eliminates all interpersonal elements of VRET. One study used virtual therapist-guided VRET, comparing it to waitlist (48). The virtual therapist used voiceover to outline principles of exposure, provide instructions and to coach participants through exercises. However, sessions were conducted in-person and selection of scenes was controlled by a researcher. Findings showed that this mode of delivery can reduce social anxiety significantly compared to a waitlist control. These promising findings add further support to the use of consumer technology in VRET.

Using automated self-guided treatments may be particularly appropriate in the case of stuttering, especially for integrating into speech therapy. This technique would cut the additional training a speech therapist requires, making it an efficient way of delivering psychological treatment alongside speech therapy. Findings from one trial demonstrate that automated CBT can aid the long-term maintenance of speech outcomes when integrated into speech restructuring (22). However, findings regarding social anxiety and fear of negative evaluation were less conclusive. Another study adopting the same automated version of CBT found it was equally as effective as *in vivo* CBT at reducing social anxiety levels (23). The benefit of self-guided VRET in this context is that behavioral treatment can be prescribed and completed remotely.

However, self-guided VRET could also work as a standalone treatment that is not delivered alongside speech therapy or prescribed by it. For some, the intensive nature of speech therapy can be a barrier to accessing psychological treatment simultaneously (96). Self-guided VRET might also be particularly relevant for those who do not have a diagnosis of SAD but have subclinical levels of social anxiety. PWS with subclinical levels of social anxiety will not typically receive clinical treatment, and they might not approach anyone regarding their anxiety. However, they may still benefit from psychological treatment, and self-guided VRET which can be delivered remotely and administered by themselves may be appropriate. Nevertheless, PWS also report that a pre-existing therapist relationship facilitates accessing psychological treatment alongside speech therapy (96). Therapeutic alliance has also been shown to be influential for speech therapy outcomes (97). Further research is therefore required to understand whether self-guided VRET is suitable and effective for PWS, and whether therapeutic alliance can be achieved with a virtual therapist.

### CBT vs. Standalone Exposure

Earlier VRET research investigated the efficacy of VRET when included into a wider CBT protocol (50–52, 56–58). All of these studies except one (56) compared the VR version of CBT to CBT using *in vivo* or imaginal exposure. Findings suggest that VR and *in vivo* CBT are largely comparable, however some evidence points to non-VR CBT treatments as more effective long-term (51, 57). It is difficult though to conclude the efficacy of VRET itself from these findings as its effects cannot be separated from the effects of cognitive treatment elements.

Delivering VRET as a standalone treatment is another way of streamlining treatment to make it less resource-heavy and more accessible. Several studies have eliminated the cognitive elements of treatment in their VRET protocols (46, 47, 53, 54). As such, the number of sessions they included was generally lower, ranging from 1 to 10 sessions. Only one of these studies compared VRET against another treatment format, finding that VRET was comparable to *in vivo* exposure at reducing social anxiety (54). However, findings also showed VRET did not reduce fear of negative evaluation, whilst *in vivo* treatment did. More recent findings show standalone VRET can reduce fear of negative evaluation (46, 47), suggesting this is not an issue for exposure conducted in VR.

However, no studies have compared standalone VRET to CBT with VRET, making it difficult to decipher the influence of cognitive elements of treatment conducted in a VR context. Previous findings suggest standalone exposure is comparable to CBT when delivered *in vivo* (26). However, authors from one of the analyzed studies make the suggestion that targeting cognitions may be more important in the context of VR, given users can make use of cognitive avoidance strategies [e.g., “the virtual social world is not real so I do not need to be afraid”; (54)]. The current findings outlined above suggest that VRET does have the potential for delivery as an effective standalone treatment, but it is not possible to conclude how its efficacy compares to virtual delivery of CBT.

## Discussion

The aim of this scoping review was to investigate design considerations for VRET protocols targeting social anxiety associated with stuttering, given the lack of research in this area. We reviewed 12 studies using VRET to reduce social anxiety, in order to understand key distinctions in treatment protocols. Based on the findings of the scoping review, we discussed how VRET could be adapted to stuttering, and informed suggestions for future research in this area.

As our findings indicate, there are several notable distinctions in how treatment protocols are designed. The choice of treatment features and protocol design will likely depend on the circumstance. For example, VRET for public speaking anxiety has shown effectiveness in reducing social anxiety levels, whereas a protocol targeting broader social fears will be more appropriate for someone experiencing general social anxiety. Similarly, the emotional processing and inhibitory learning approaches to exposure have both shown success in VRET, and each will be more suitable in particular contexts. Whilst inhibitory learning techniques are principally suited to phasing out safety behaviors and treating anxiety across a variety of contexts, the emotional processing approach is far simpler to administer, making it well-suited for automated VRET. The other notable finding is the adoption of new technology. Recent advances in 360° video quality have made it possible to create virtual environments with greater ease. Additionally, standalone and smartphone HMDs have progressed the development of automated VRET guided by a virtual therapist. The benefit of these various approaches is that VRET can be adapted for different contexts.

PWS may benefit from some of the protocols examined in the reviewed studies but adapting VRET to stuttering-specific fears will ensure it considers the unique experience of comorbid stuttering and social anxiety. However, a general limitation of VRET is the difficulty of personalizing exercises to individual fears, as exercises must be created in advance. Exposure exercises should be adapted to target key stuttering-related themes such as telephone speaking and word substitution, but individual fears, safety behaviors and beliefs will vary. Nevertheless, we believe several VRET techniques adopted in previous protocols could be used in VRET designed for PWS. The inhibitory learning method may be appropriate for integrating individual expectations and safety behaviors into exposure exercises. This approach might also be particularly suitable for targeting reacquisition of fear, which is a greater risk for PWS considering the continued reactions from others in relation to speech. Given the challenges of adapting VRET to stuttering and the varied experiences of social anxiety across this group, it is key that PWS are involved in the design process to ensure maximum relevance.

Of particular interest to stuttering-specific VRET is automation and the use of virtual therapists. This may be a more approachable technique for individuals whose primary fears revolve around communication and social evaluation—fears which may arise in the presence of a therapist (98). This could also be suitable for PWS who experience subclinical social anxiety. However, development of self-guided VRET is in its early stages and only a handful of studies have adopted virtual therapists for social anxiety (48) and other types of anxiety (99–101). Further research is required to explore the applicability of these techniques. Successfully recreating the therapist role will be integral to remote delivery of VRET, and therapeutic alliance is typically an influential factor in other stuttering treatments. Whilst some evidence supports the therapeutic relationship in VRET (50, 102), no study has explored whether similar levels of therapeutic alliance can be achieved with a virtual therapist. As highlighted previously, the relationship between therapeutic alliance and treatment outcome is not straightforward for social anxiety (52, 90, 91). However, given that virtual therapist-guided VRET eliminates the human social exchange, therapeutic alliance might be bolstered in this format. More research is required to explore whether these techniques are suitable and effective for PWS as well as the influence of different embodiments of the virtual therapist.

Another suggestion is to include VRET in speech therapy. Whilst this approach might not yield benefits to social anxiety, it is expected to aid retention of benefits from speech therapy and increase the uptake of social anxiety treatment amongst PWS. Future research could also target the intertwined issues associated with comorbid stuttering and social anxiety by integrating VRET and Acceptance and Commitment Therapy (ACT). Whilst exposure therapy focuses on eliminating or inhibiting fear and distressing thoughts, ACT targets the consequent struggle to control or eliminate these thoughts. Symptom reduction is therefore not a primary aim of ACT; rather, it encourages the patient to fully experience and embrace anxiety by teaching acceptance of these thoughts and feelings (103). For PWS, anxiety revolves around their stutter and negative self-perceptions of their speech (11), which contributes to greater speech dissatisfaction (12). Treatments which promote self-efficacy are also important for long-term improvement (104). When combined with VRET, ACT may aid treatment engagement and reduce safety behaviors (105), whilst targeting the broader experience of negative thoughts surrounding stuttering. Research supports the use of ACT to aid maintenance of benefits from speech therapy and reduce the adverse impact of stuttering on participants' lives (106). Findings from two other studies support the use of ACT in the wider population to reduce social anxiety, demonstrating equal effectiveness with CBT (107, 108). Research has already experimented with combining VRET and ACT for treating social anxiety amongst fluent speakers, suggesting this might be a suitable technique for reducing social anxiety (109). More research is required to understand the contribution of ACT to treatment outcome when combined with VRET, and how such a combination of treatments compares to existing social anxiety treatments. Lastly, research should also seek to understand whether this can be effective in PWS.

More research is also required to understand the processes that make VRET effective. Presence is another concept that is theorized to have a significant influence on treatment outcome and experiencing anxiety virtually. Whilst presence appears to be a strong predictor of anxiety for most anxiety disorders, it is not clear whether this relationship exists at all for SAD (34, 110, 111). It is suggested that the constructs that make up presence may be less relevant to experiencing fear of negative evaluation, which is triggered by social cues. As such, presence may also have less of an impact on treatment effectiveness and symptom change (87). Attention has also turned to social presence, which is likely to play a more significant role within social virtual environments. Social presence is conceptually distinct from physical presence (112) and describes the sensation of being in the presence of another being, which requires a level of cognitive and emotional engagement. No study to date has investigated the relationship between social presence and VRET outcomes, but SAD patients have shown heightened co-presence and mutual attention in response to virtual environments (33), suggesting it might be influential. These links require further investigation to inform the treatment features and modalities necessary for treating social anxiety.

### Review Limitations

There are several limitations to the current scoping review. First, as we did not conduct a meta-analysis or any other quantitative analysis, we cannot directly compare the effectiveness of different treatments and the techniques they use. A scoping review approach was chosen based on the relatively small literature on VRET protocols for social anxiety and PWS, and to provide a qualitative overview of these protocols. Second, there were inconsistencies and missing details in the reporting of VRET protocols, study methods and findings. As a result, there may have been details that were missed in the analyses. This is a common limitation, however, and points to the importance of creating standardized reporting methods for interventions contributing to reproducibility as well as replicability of findings in this area. Third, several studies investigating VRET for social anxiety were not included in the review as they were outside of the scoping criteria. Some of these expand on innovative techniques such as cloud-based VRET (113), or broaden our understanding of VRET effectiveness by using functional magnetic resonance imaging (114), yet the cumulative effects of such protocols are still unknown due to their recency. Finally, this review focused on adults due to differences in developmental trajectories of social anxiety as well as limited availability of protocols for children and adolescents. Nonetheless, more research is required on the usefulness and efficacy of VRET for non-adult populations.

### Review Strengths

This scoping review represents the first comprehensive overview of the various techniques and approaches used in VRET protocols targeting social anxiety. To our knowledge, this is also the first scoping review to focus on exposure protocols designed for PWS. Our findings provide a deeper understanding of the comparative value of different VRET approaches, and how these can be used to suit different circumstances. Furthermore, all reviewed studies compared VRET against another treatment or control condition, allowing greater confidence in the conclusions drawn about the efficacy of different protocols.

## Conclusion

The past decade has seen an increased interest in using virtual reality for mental healthcare. This has coincided with the rising availability of commercial VR headsets, paired with overwhelmed mental healthcare systems across the globe. Yet there is much work to be done regarding systematic understanding of VR intervention protocols, specifically for certain groups such as PWS. Due to a lack of research on the suitability of VRET for PWS, it is difficult to draw conclusions about the effectiveness of these techniques. The current review suggests several techniques that may be appropriate in the case of stuttering that need further testing, including automated VRET using a virtual therapist, inhibitory learning techniques, and integration with other treatments. Regardless of the approach taken, it is key that VRET is adapted to the nature of social anxiety associated with stuttering. Finally, our review has also highlighted the need for creating, sharing, and testing VR intervention protocols in a systematic manner, to increase reproducibility as well as replicability of findings.

## Author Contributions

IC: conceptualization, literature search and analysis, and writing original draft. IC and NZ: writing reviews and editing. NZ: supervision. Both authors contributed to the article and approved the submitted version.

## Funding

The research was funded by a training grant from UK Research and Innovation and Imperial College London (no. EP/R513052/1).

## Conflict of Interest

The authors declare that the research was conducted in the absence of any commercial or financial relationships that could be construed as a potential conflict of interest.

## Publisher's Note

All claims expressed in this article are solely those of the authors and do not necessarily represent those of their affiliated organizations, or those of the publisher, the editors and the reviewers. Any product that may be evaluated in this article, or claim that may be made by its manufacturer, is not guaranteed or endorsed by the publisher.

## Abbreviations

VRET: virtual reality exposure therapy
PWS: people who stutter
SAD: social anxiety disorder
CBT: cognitive behavioral therapy
VR: virtual reality
HMD: head-mounted display
ACT: acceptance and commitment therapy.
