# Supplementary material for: Virtual Reality Exposure Therapy for Treating Social Anxiety: A Scoping Review of Treatment Designs and Adaptation to Stuttering
Source: Front Digit Health. 2022 Feb 25;4:842460. doi: 10.3389/fdgth.2022.842460 (PMC8913509; doi:10.3389/fdgth.2022.842460)
Supplement: Supplementary file 1 [file Data_Sheet_1.docx]

Supplementary Material

Appendix A. Database search strategy

**PsycINFO/PsycARTICLES**

Search 1

1. (virtual reality or VR).mp. [mp=ti, ab, tx, ct, hw, tc, id, ot, tm, mh]
2. limit 1 to english language
3. limit 2 to full text
4. (therapy or treatment).mp. [mp=ti, ab, tx, ct, hw, tc, id, ot, tm, mh]
5. limit 4 to english language
6. limit 5 to full text
7. (social anxiety or social phobia or social anxiety disorder or SAD).mp. [mp=ti, ab, tx, ct, hw, tc, id, ot, tm, mh]
8. limit 7 to english language
9. limit 8 to full text
10. 3 and 6 and 9
11. remove duplicates from 10

Search 2

1. (virtual reality or VR).mp. [mp=ti, ab, tx, ct, hw, tc, id, ot, tm, mh]
2. limit 1 to english language
3. limit 2 to full text
4. (therapy or treatment).mp. [mp=ti, ab, tx, ct, hw, tc, id, ot, tm, mh]
5. limit 4 to english language
6. limit 5 to full text
7. (stutter$ or stammer$).mp. [mp=ti, ab, tx, ct, hw, tc, id, ot, tm, mh]
8. limit 7 to english language
9. limit 8 to full text
10. 3 and 6 and 9
11. remove duplicates from 10

Search 3

1. (stutter$ or stammer$).mp. [mp=ti, ab, tx, ct, hw, tc, id, ot, tm, mh]
2. limit 1 to english language
3. limit 2 to full text
4. (therapy or treatment).mp. [mp=ti, ab, tx, ct, hw, tc, id, ot, tm, mh]
5. limit 4 to english language
6. limit 5 to full text
7. exposure.mp. [mp=ti, ab, tx, ct, hw, tc, id, ot, tm, mh]
8. limit 7 to english language
9. limit 8 to full text
10. 3 and 6 and 9
11. remove duplicates from 10
